# Elucidating the mechanisms and mitigation strategies for six-phthalate-induced toxicity in male germ cells

**DOI:** 10.3389/fcell.2024.1398176

**Published:** 2024-07-10

**Authors:** Seok-Man Kim, Yong-Hee Kim, Gil Un Han, Seul Gi Kim, Bang-Jin Kim, Sung-Hwan Moon, Seung Hee Shin, Buom-Yong Ryu

**Affiliations:** ^1^ Department of Animal Science and Technology, Chung-Ang University, Anseong-Si, Gyeonggi-Do, Republic of Korea; ^2^ AttisLab Inc., Anyang-Si, Gyeonggi-Do, Republic of Korea; ^3^ Department of Surgery, Division of Surgical Sciences, Columbia University Irving Medical Center, New York, NY, United States

**Keywords:** phthalate mixture, GC-1 spermatogonia, apoptosis, autophagy, mitigation strategy

## Abstract

Phthalate esters (PAEs) are primary plasticizers and endocrine-disrupting chemicals (EDCs) that are extensively used in numerous everyday consumer products. Although the adverse effects of single PAEs have been studied, our understanding of the effect of multiple phthalate exposure on male germ cell vitality remains limited. Therefore, this study aimed to investigate the collective effects of a mixture of PAEs (MP) comprising diethyl-, bis (2-ethylhexyl)-, dibutyl-, diisononyl-, diisobutyl-, and benzyl butyl-phthalates in the proportions of 35, 21, 15, 15, 8, and 5%, respectively, on differentiated male germ cells using GC-1 spermatogonia (spg) cells. As a mixture, MP substantially hindered GC-1 spg cell proliferation at 3.13 μg/mL, with a half-maximal inhibitory concentration of 16.9 μg/mL. Treatment with 25 μg/mL MP significantly induced reactive oxygen species generation and promoted apoptosis. Furthermore, MP activated autophagy and suppressed phosphorylation of phosphoinositide 3-kinase, protein kinase B, and mammalian target of rapamycin (mTOR). The triple inhibitor combination treatment comprising parthenolide, N-acetylcysteine, and 3-methyladenine effectively reversed MP-induced GC-1 spg cell proliferation inhibition, mitigated apoptosis and autophagy, and restored mTOR phosphorylation. This study is the first to elucidate the mechanism underlying MP-induced male germ cell toxicity and the restoration of male germ cell proliferation mediated by chemical inhibitors. Therefore, it provides valuable insights into the existing literature by proposing a combinatorial toxicity mitigation strategy to counteract male germ cell toxicity induced by various EDCs exposure.

## Introduction

Plastics are integral to modern life, representing one of the most extensively manufactured synthetic materials. Of the additives employed in plastic production, phthalate esters (PAEs) play an important role as plasticizers, used widely in diverse products ranging from children’s toys to medical equipment (Casals-Casas and Desvergne, 2011; Katsikantami et al., 2016). However, certain high-molecular-weight PAEs, notably di (2-ethylhexyl) phthalate (DEHP), serve as endocrine-disrupting chemicals (EDCs), implicated in a spectrum of human health issues encompassing developmental, reproductive, neurological, and immune system dysfunction (Monneret, 2017). The pervasive presence of PAEs in the environment, coupled with limited disclosure of product ingredients, poses challenges for conscious avoidance (Dodson et al., 2012). As a result, humans experience considerable PAE absorption through various routes, including food consumption, inhalation, and skin contact leading to detectable levels of PAEs in the human body (Benjamin et al., 2017) and associated health complications such as allergic reactions, asthma, and cardiovascular and kidney diseases (North et al., 2014; Mariana et al., 2016; Malits et al., 2018). In the context of reproductive effects, certain PAEs, as EDCs, can disrupt the male reproductive system and crucial developmental functions essential for generational continuity (Fisher, 2004; Boberg et al., 2008; Kay et al., 2014; Radke et al., 2018). Additionally, PAEs can adversely affect female reproductive health by altering ovarian follicle growth and hormone levels. A mixture of PAEs (MP) can even cause multi- and transgenerational effects on the female reproduction system (Zhou et al., 2017a; Zhou et al., 2017b; Alejandra et al., 2024; Santacruz-Márquez et al., 2024).

Male fertility fundamentally relies on spermatogenesis, a lifelong process that commences during embryonic development. Throughout spermatogenesis, undifferentiated male germ cells, spermatogonia (spg), undergo the multifaceted processes of mitosis and meiosis to produce spermatozoa within the seminiferous tubules of the testis (Brinster, 2007). During this intricate process, various factors, such as hormones and microenvironmental conditions within the testes, are critical for the development of male germ cells (Hofmann, 2008; Hai et al., 2014). Numerous studies have utilized *in vitro* model systems to explore the endocrine-disrupting effects of PAEs on the testicular microenvironment, encompassing various cell types at the cellular level. For instance, PAEs have been demonstrated to trigger apoptosis and autophagy in Sertoli and Leydig cells, which are pivotal in facilitating gonadal cell growth and the testosterone production, both crucial for spermatogenesis (Shima et al., 2013; Wang et al., 2017; Sun et al., 2018). Additionally, studies have demonstrated the adverse effects of PAEs in GC-1 spg cells (Li et al., 2019; Gan et al., 2020), which represent a male germ cell line situated between type B spermatogonia and primary spermatocytes (Hofmann et al., 1992). Cells in this stage undergo two successive meiotic divisions, ultimately contributing to spermatogenesis and male fertility by generating spermatids (Bharath, 2017). While the detrimental effects of several single PAEs have been reported in various cell types (Wójtowicz et al., 2017; Tripathi et al., 2019; Wang et al., 2023), exposure to PAEs typically occurs through a mixture. Commonly used PAEs include diethyl phthalate (DEP), DEHP, di-n-butyl phthalate (DBP), diisononyl phthalate (DiNP), diisobutyl phthalate (DiBP), butyl benzyl phthalate (BBzP), and diisodecyl phthalate (DiDP) (Johns et al., 2015). As a result, these PAEs are often detected in a mixed form in human urine (Koch et al., 2017; Zhou and Flaws, 2017).

Urinary metabolites serve as a comprehensive indicator of cumulative exposure, making monitoring human biomarkers and measuring specific metabolites in urine an optimal method for assessing total phthalate exposure in individuals (Koch et al., 2007; Koch and Calafat, 2009). Therefore, addressing the complex impacts of PAEs as EDCs in a mixed formulation is the most suitable approach to mimic the uncontrolled and simultaneous effects of PAEs on human health. In this regard, studies have demonstrated that the effects of MP, consisting DEP, DEHP, DBP, DiNP, DiBP, and BBP, can lead to postnatal reproductive tract malfunctions in male mice (Barakat et al., 2019). Another study revealed that MP, comprising BBP, DEP, DEHP, DiBP, and dipentyl phthalate, disrupted testicular steroidogenesis in rats (Howdeshell et al., 2015). However, the comprehensive molecular mechanisms underlying MP’s direct effects on male germ cells remain unclear.

Therefore, the aim of the current study was to investigate the detrimental effects of MP on male germ cells using GC-1 spg cells, elucidate the pathways targeted by MP toxicity, and address scenarios of MP exposure. Furthermore, the study demonstrated protective therapeutic measures against the toxicity induced by multiple PAEs. Elucidating the mechanism of MP toxicity and identifying effective inhibitors provides valuable insights into male germ cell toxicity caused by environmental factors and aids in developing potential therapeutic interventions to protect male germ cells from exposure to multiple PAEs.

## Materials and methods

### Cell culture and preparation of MP

The GC-1 spg cell line (CRL-2053, ATCC, Virginia, United States) was cultured in Dulbecco’s modified Eagle’s medium (DMEM, L0103-500, Biowest, Nuaillé, France) containing 10% fetal bovine serum (FBS, US-FBS-500, GW Vitek, Seoul, Korea) and 1% penicillin and streptomycin (15140122, Gibco, Thermo Fisher Scientific, Waltham, MA, United States) in an incubator maintained at 5% CO_2_ and 37°C. For exposure to MP, phenol red-free DMEM (LM001-10, WELGENE, Gyeongsan, Korea) supplemented with 1% FBS and 1% penicillin/streptomycin (defined as the basal medium in this study) was used to minimize the impacts of growth factors and hormones included in FBS (Welshons et al., 1988).

To prepare the MP, we combined DEP (CAS 84-66-2), DEHP (CAS 117-81-7), DBP(CAS 84-74-2), DiNP (CAS 28553-12-0), DiBP (CAS 84-69-5), and BBP (CAS 85-68-7) (all PAEs were obtained from Sigma-Aldrich, St. Louis, MO, United States) in proportions of 35%, 21%, 15%, 15%, 8%, and 5%, respectively, following the ratios described in previous research (Zhou and Flaws, 2017; Amjad et al., 2021). This high-concentration stock mixture solution was then diluted using dimethyl sulfoxide (DMSO, D2650, Sigma-Aldrich) to achieve the desired PAE concentration (25 mg/mL) in the MP. Serial dilutions were made using this 25 mg/mL MP in a 1:2 ratio to prepare the final working concentrations of 0 (vehicle control, consisting only of 0.1% DMSO in basal medium), 1.56, 3.13, 6.25, 12.5, and 25 μg/mL in the basal medium.

For MP treatment, GC-1 spg cells were seeded at a density of 7.0 × 10^4^ cells per well in a six-well plate and incubated for 24 h. Subsequently, the cells were treated with MP and incubated for another 48 h.

### MP-induced cytotoxicity assessment

To evaluate the cytotoxic effects of MP on GC-1 spg cells, the cells were cultured and treated with MP as described. The GC-1 spg cells exposed to MP were harvested using 0.25% trypsin-ethylenediaminetetraacetic acid (1×; EDTA, 25200-072, Gibco, Grand Island, NY, United States), then centrifuged for 6 min at 600 × *g* at 4°C. The resulting cell pellet was resuspended in basal medium. A 10 µL aliquot of cells suspended in basal medium was mixed with 10 µL of trypan blue solution (15250-061, Gibco). A volume of 10 µL of the mixed suspension was loaded into a hemocytometer, and unstained (live) and stained (dead) cells were counted under a microscope. The proliferation rates were calculated using the following equations:

$$\text{Proliferation rate }\left(\%\right)=\frac{\text{number}\;\text{of}\;\text{harvested}\;\text{cells}\;\text{treated}\;\text{with}\;\text{MP}}{\text{number}\;\text{of}\;\text{initial}\;\text{cells}\;\text{per}\;\text{well}\;\text{plated}}\times 100$$

$$\text{Relative proliferation rate }\left(\%\right)=\frac{\text{proliferation}\;\text{rate}\;\text{of}\;\text{each}\;\text{MP}\;\text{treatment}\;\text{group}}{\text{proliferation}\;\text{rate}\;\text{of}\;\text{the}\;\text{control}\;\text{group}}\times 100$$

The half maximal inhibitory concentration (IC_50_) was determined, as previously reported (Toma et al., 2017).

### Detection of apoptosis using fluorescence-activated cell sorting (FACS)

To assess whether MP induced apoptosis in GC-1 spg cells, an annexin V conjugated to allophycocyanin (APC) apoptosis kit (559763, BD Biosciences, San Jose, CA, United States) was utilized. Briefly, cell pellets from both the 0 and 25 μg/mL MP-exposed groups were collected using 0.25% trypsin-EDTA (1×), then centrifuged for 6 min at 600 × *g* at 4°C. Subsequently, the cells from each group were counted and resuspended in 1 × binding buffer (51-66121E, BD Pharmingen^TM^, San Diego, CA, United States) to a concentration of 1.0 × 10^6^ cells/mL. The cell suspension was aliquoted and incubated with annexin V-APC and propidium iodide (PI, P4170, Sigma-Aldrich) for 15 min at room temperature (RT; 20°C–25°C) in the dark. Before analysis, 400 µL of 1 × binding buffer was added to the control and treatment groups. The annexin V assay was performed using the FACS Aria Ⅱ cell sorter (BD Biosciences) equipped with BD FACS Diva software (Version 6.1.3, BD Biosciences).

### Western blot analysis

In this study, all experimental groups were isolated using Totex lysis buffer (Teo et al., 2010; Shin et al., 2014) on ice, with intermittent vortexing of the lysate every 5 min for 30 min. After centrifugation at 17,950 × *g* (Combi-514R, Hanil, Korea) for 30 min at 4°C, the supernatants containing soluble proteins were collected. Total protein concentration was determined using the Bradford assay (5000006, Bio-Rad, Alfred Nobel Drive Hercules, CA, United States), and equal quantities of protein lysate were loaded onto 6%–15% tris-glycine polyacrylamide gels. Proteins were then transferred onto methanol-activated polyvinylidene difluoride membranes (IPVH00010, Millipore, Billerica, MA, United States). Non-specific binding was blocked with a 5% skim milk solution in Dulbecco’s phosphate buffered saline (DPBS, 14200-075, Gibco) containing 0.1% Tween 20 (PBS-T) for 1 h at RT. After washing the membrane three times with PBS-T, it was incubated with primary antibody overnight with gentle shaking at 4°C in the dark. Secondary antibody staining was conducted for 2 h at RT. Following three times PBS-T washes, protein visualization was achieved using two electro-chemiluminescence Pico or Femto-ECL systems (Clarity™ Western ECL Substrate, Bio-Rad). The membrane images were captured using Touch Imager (e-BLOT Life Science, Shanghai, China), and protein band intensity was normalized using ImageJ software (version 1.8.0, National Institutes of Health, Bethesda, MD, United States) for comparison between control and MP treatment groups. Antibodies used are listed in Supplementary Table S1, and intensity data were validated against the loading control (α-tubulin) in all independent experiments (*n* = 3).

### Reactive oxygen species (ROS) detection using 2′, 7′-dichlorofluorescein diacetate (DCFDA) fluorescence microscopy

To assess MP-triggered ROS production, we utilized a DCFDA (287810, Millipore) probe as described (Figueroa et al., 2018) and compared the control and MP-exposed groups. Briefly, GC-1 spg cells were plated in six-well plates at a concentration of 7.0 × 10^4^ cells per well for both control and MP-exposed groups. After 24 h of incubation, cells were exposed to DMSO or MP for 48 h. Following this, cells from each group were rinsed with DPBS supplemented with 1% FBS (PBS/FBS) and then stained with 10 μM DCFDA in basal medium for 45 min at 37°C. After incubation, cells were washed two times with PBS/FBS. Each group was subsequently counterstained with 4 μg/mL Hoechst 33342 (B2261, Sigma-Aldrich) at 37°C for 10 min to visualize cell nuclei. After washing with cold PBS/FBS, fluorescence images were captured using a confocal microscope (LSM800Airy, Carl Zeiss, Oberkochen, Germany). The percentage of dichlorofluorescein (DCF)-positive cells was calculated from three separate experiments as follows:

$${\text{DCF}}^{+}\;\left(\%\right)=\text{the number of DCF}-\text{positive cells}/\text{Hoechst }33342-\text{stained cells}$$

### Acridine orange (AO) staining for autophagy detection

AO (158550, Sigma-Aldrich) staining was performed to visualize the MP-induced autophagic activity. GC-1 spg cells were seeded in a confocal dish for 24 h and treated with MP for 48 h. Afterward, cells were rinsed once with PBS/FBS and incubated with 1 μg/mL of AO at 37°C for 15 min. Following incubation, AO-stained cells were washed two times with PBS/FBS and labeled with Hoechst 33342 dye for 10 min at 37°C to visualize the nuclei. After a single wash with cold PBS/FBS, images of AO (red and green fluorescence) and Hoechst 33342 fluorescence were captured using a confocal microscope. The red-to-green intensity ratio (R/GFIR) was calculated as:

R/GFIR=Intensity of red fluorescence per cell/Intensity of green fluorescence per cell

$$\text{Relative level of }R/\text{GFIR }\left(\text{fold change}\right)=R/\text{GFIR of MP}-\text{treated cells}/R/\text{GFIR of control cells}$$

### Evaluation of inhibitor combination treatment

To alleviate MP-derived toxicity, three inhibitors were sourced: parthenolide (PTL, an NF-κB inhibitor, 512732, Sigma-Aldrich), 3-methyladenine (3-MA, an autophagy inhibitor, 3977, Tocris Bioscience, Bristol, United Kingdom), and N-acetylcysteine (NAC, a ROS inhibitor, A7250, Sigma-Aldrich). PTL and 3-MA were dissolved in DMSO and NAC was dissolved in the basal medium to reach the following desired final concentrations: PTL (1 μM), 3-MA (0.1 μM), and NAC (1 mM). The concentration range of the inhibitors was based on previous studies (Li-Weber et al., 2005; Xiao et al., 2016; Jung et al., 2022; Kim et al., 2024). To address potential antagonism when combining the inhibitors, concentrations were set lower than those typically used individually. Alleviation assessment involved comparing the control (0 μg/mL) and MP-exposed group (25 μg/mL) with the corresponding inhibitor co-treatment groups. Each inhibitor’s ability to mitigate MP-induced GC-1 spg cell toxicity was evaluated. Cells for each treatment set were pre-treated with the inhibitors in a six-well plate and incubated for 24 h. Then, the medium of the control group was replaced with inhibitors only, and the MP with inhibitors, followed by an additional incubation for 48 h. Subsequently, the effects of single, dual, and triple inhibitors were analyzed using the proliferation assay (see Section 2.2) and western blotting (see Section 2.4).

### Statistical analyses

Statistical analyses were performed using Prism software (version 8.0.1; GraphPad Software, La Jolla, CA, United States). Data are presented as mean ± standard error of the mean. For proliferation analysis, one-way analysis of variance (ANOVA) and Dunnett’s test compared control and MP-exposed groups. Student’s t-test was conducted to determine significant differences in the DCFDA assay, annexin V/PI data using FACS, and western blotting. Combination inhibitor treatment experiments were analyzed using One-way ANOVA and Tukey’s honestly significant difference *post hoc* test. Significant differences are denoted by symbols: * represents a significant difference between the control and MP-exposed groups, and # indicates a significant difference between MP-exposed groups without inhibitors and those co-treated with inhibitors. The significance threshold was set at *p* < 0.05. Experiments were independently performed at least three times, and the number of replicates is denoted in the figure legends.

## Results

### Evaluation of a mixture of PAEs (MP)-induced GC-1 spg cell toxicity

**FIGURE 1:**
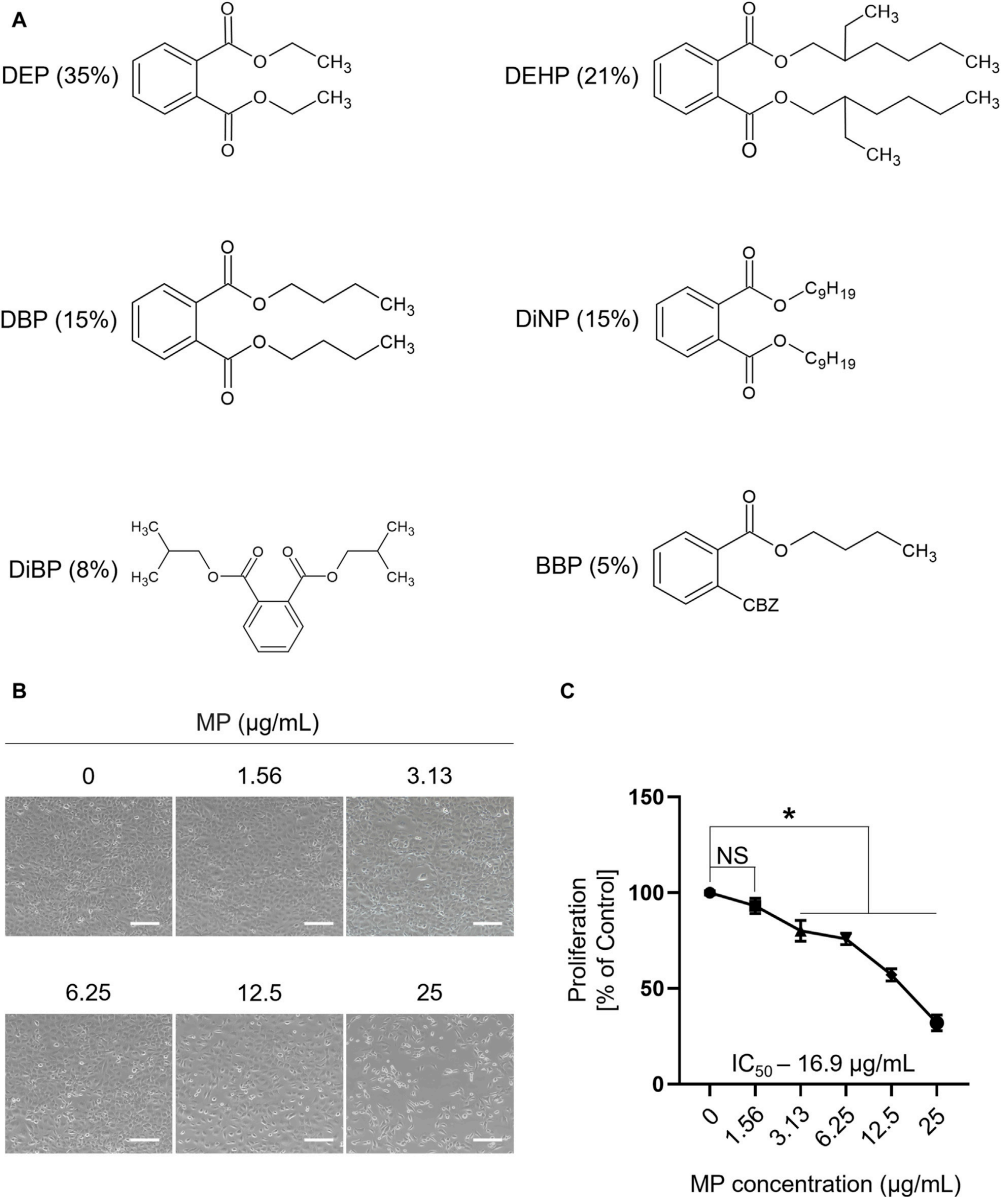
A mixture of PAEs (MP) inhibits the proliferation of GC-1 spg cells. **(A)** Chemical structure and percentage of the six phthalate esters in MP. **(B)** Representative microscopic images of GC-1 spg cells exposed to varying concentrations of the mixture for 48 h (scale bar = 200 μm). **(C)** The proliferation rate (mean ± SEM) of GC-1 spg cells is shown. One-way ANOVA was used for statistical analysis and multiple comparisons were done using Dunnett’s test. Significant differences between each group are denoted by an asterisk (*p < 0.05, *n* = 5). PAEs, phthalate esters; spg, spermatogonia; DEP, diethyl phthalate; DEHP, bis(2-ethylhexyl) phthalate; DBP, dibutyl phthalate; DiNP, diisononyl phthalate; DiBP, diisobutyl phthalate; BBP, benzyl butyl phthalate; ANOVA, analysis of variance; IC_50_, half maximal inhibitory concentration; SEM, standard error of the mean; NS, non-significant.

The chemical structures and composition of MP are shown in Figure 1A. Cytotoxic effects of MP on GC-1 spg cells were determined across various concentrations using a trypan blue exclusion assay. A notable decrease in GC-1 spg cell proliferation was evident at 3.13 μg/mL MP, indicating a concentration-dependent proliferation inhibitory effect. This trend was corroborated by the sulforhodamine B assay (Figures 1B, C; Supplementary Figure S1). The IC_50_ concentration was determined as 16.9 μg/mL, falling between 12.5 μg/mL and 25 μg/mL. Although 12.5 μg/mL approximates the IC_50_ concentration, we opted for 25 μg/mL (near IC_70_) to explore potential adverse effects comprehensively and gain insights into MP’s impact on male germ cells. These insights included ROS generation, apoptosis and autophagy induction, and regulation of key kinase phosphorylation critical for male germ cell proliferation.

### MP triggered ROS generation and induced apoptosis in GC-1 spg cells

**FIGURE 2:**
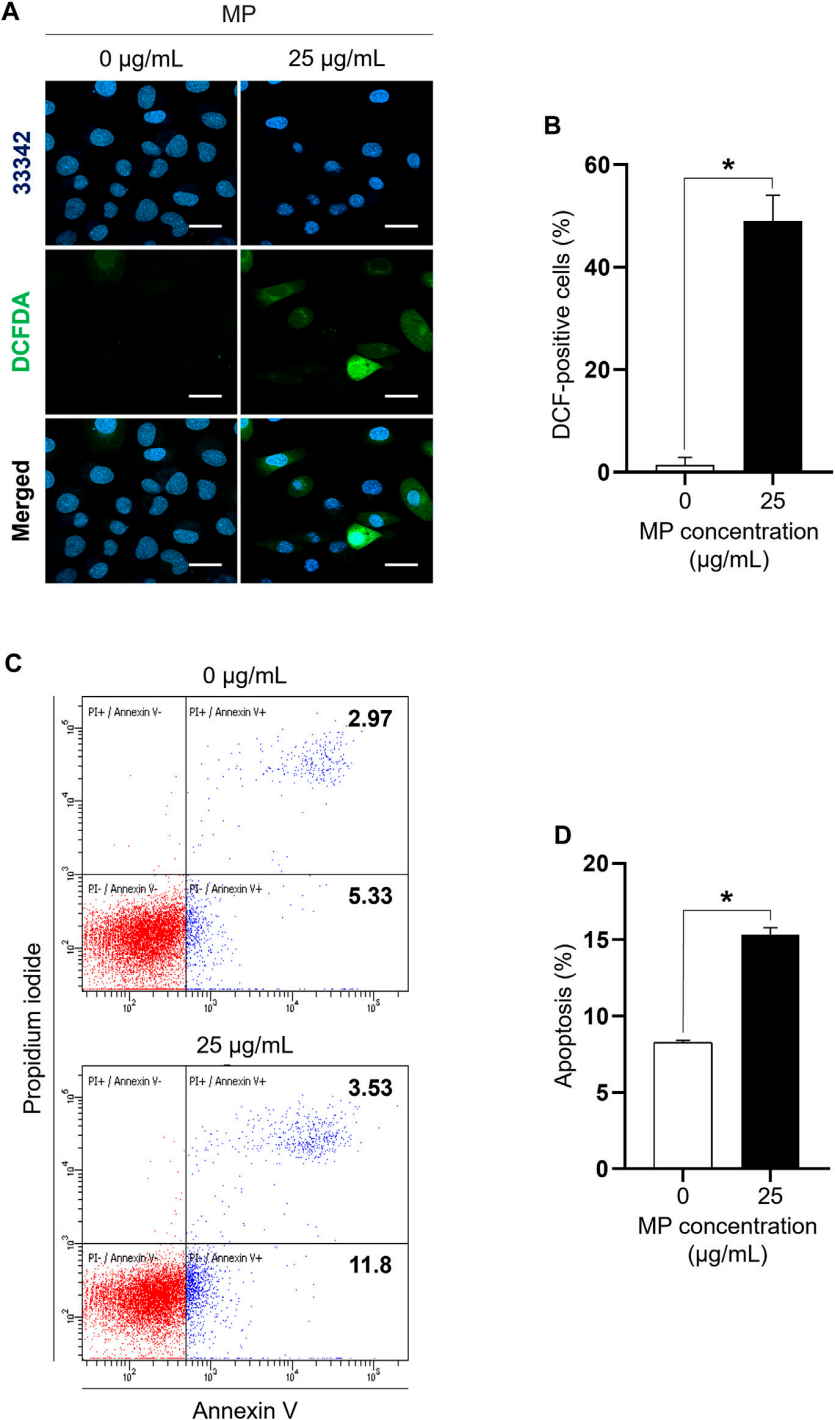
A mixture of PAEs (MP) markedly increases ROS generation and induces apoptosis in GC-1 spg cells. **(A)** Representative confocal microscope images of GC-1 spg cells exposed to 0 and 25 μg/mL MP for 48 h. Fluorescent microscopic images of DCF-positive cells oxidized by ROS are presented (Hoechst 33342 for visualizing nucleus, scale bar = 50 μm). **(B)** DCF-positive GC-1 spg cells shown in **(A)** (*n* = 3). **(C)** Dot plot images exhibiting apoptotic cells in the control and MP-exposed groups. **(D)** MP-induced apoptotic cells shown in **(C)** (*n* = 3). Graphical representations are shown as the mean ± SEM. The statistical analysis was performed using the Student’s t-test, and significant differences are indicated by an asterisk (*p < 0.05). PAEs, phthalate esters; ROS, reactive oxygen species; spg, spermatogonia; DCF, dichlorodihydrofluorescein; DCFDA, dichlorodihydrofluorescein diacetate; SEM, standard error of the mean.

DCFDA staining and annexin V assays were performed to assess MP-induced ROS generation and apoptosis in GC-1 spg cells. Strong DCF-positive staining was evident in GC-1 spg cells exposed to 25 μg/mL MP, with a significant difference observed between the MP group (49.05%) and the control group (1.45%) (Figures 2A, B). Moreover, apoptosis rates were notably higher in cells exposed to 25 μg/mL MP (15.33%) compared to control cells (8.27%) (Figures 2C, D). These findings demonstrate that MP significantly induced ROS production and activated apoptosis in GC-1 spg cells. Taken together, these results suggest that MP inhibited cell proliferation via excessive oxidative stress and apoptosis in GC-1 spg cells.

### MP activated both intrinsic and extrinsic apoptotic pathways in GC-1 spg cells

**FIGURE 3:**
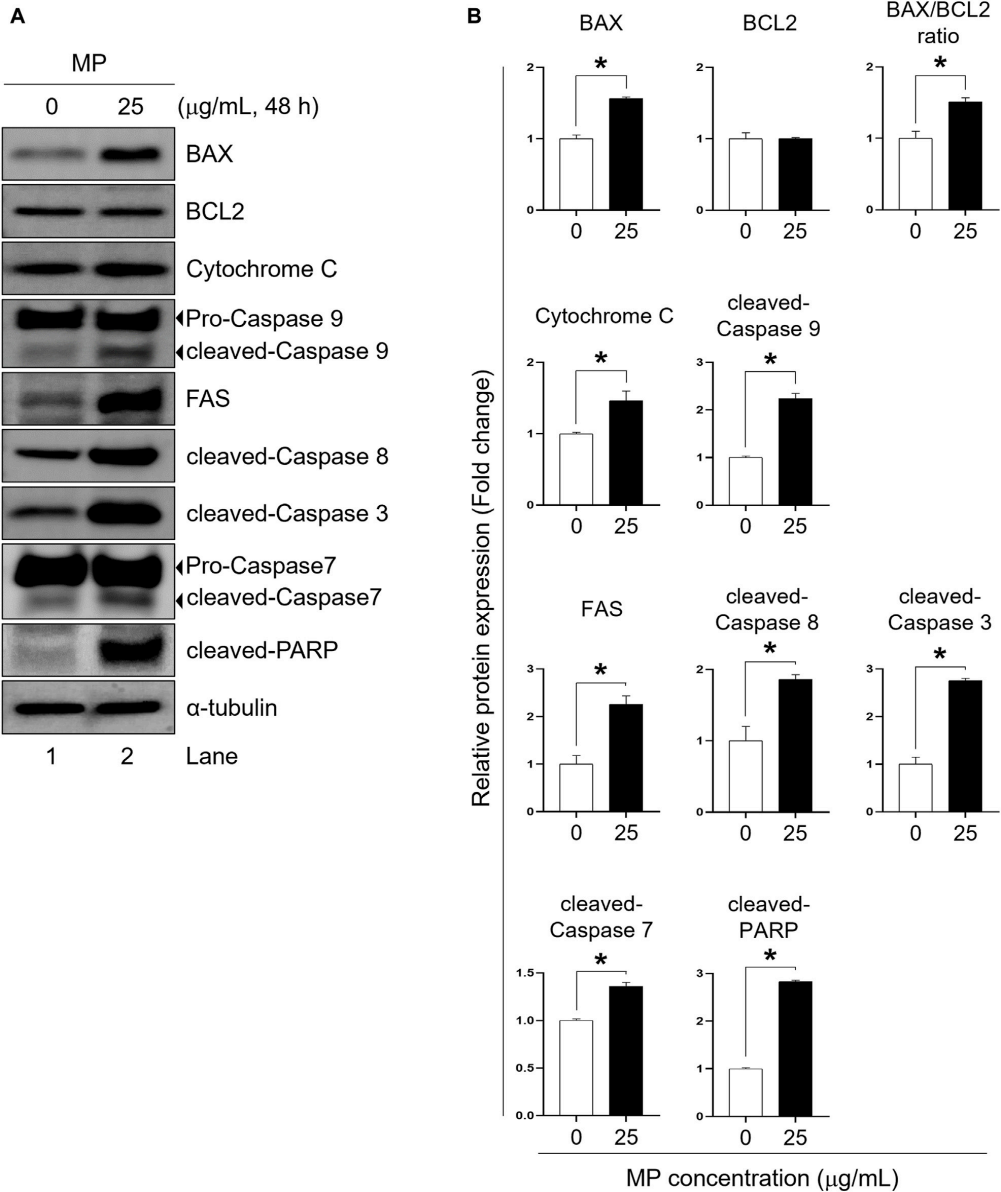
A mixture of PAEs (MP) promotes apoptotic pathways in GC-1 spg cells. **(A)** Representative western blot images displaying the protein markers of apoptotic pathways: intrinsic (BAX, BCL2, cytochrome c, and cleaved-caspase 9), extrinsic (FAS and cleaved-caspase 8), and execution proteins (cleaved-caspase 3, cleaved-caspase 7, and cleaved-PARP). **(B)** Normalized protein expressions shown in **(A)**. All values are shown as the means ± SEM. The statistical analysis was carried out using Student’s t-test. The significant differences in each group are indicated with an asterisk (*p < 0.05, *n* = 3). PAEs, phthalate esters; spg, spermatogonia; BCL2, B-cell lymphoma 2; BAX, BCL2-associated X protein; PARP, poly (ADP-ribose) polymerase; SEM, standard error of the mean.

FACS analysis revealed that MP exposure induced both early and late stages of apoptosis in GC-1 spg cells (Figures 2C, D). To elucidate the underlying mechanism of MP-induced apoptosis in GC-1 spg cells, the expressions of apoptosis regulator proteins specific to intrinsic and extrinsic apoptotic pathways were assessed. Firstly, intrinsic apoptosis markers including cytochrome c, cleaved-caspase 9, pro-apoptotic BCL2-associated X protein (BAX), and anti-apoptotic B-cell lymphoma 2 (BCL2) were examined. Compared to the control group, the 25 μg/mL MP-exposed group exhibited substantially elevated levels of BAX, cytochrome c, and cleaved-caspase 9, while BCL2 showed no marked differences at this concentration (Figures 3A, B). The BAX/BCL2 ratio notably increased in the 25 μg/mL MP-exposed group (Figure 3B), indicating active involvement in the intrinsic apoptotic pathway. Additionally, analysis of extrinsic and execution apoptosis markers revealed a significant increase in extrinsic (FAS, cleaved-caspase 8) and execution (cleaved-caspase 3, cleaved-caspase 7, cleaved-poly (ADP-ribose) polymerase [PARP]) proteins (Figures 3A, B). These findings suggest that MP induces a reduction in proliferation through both the intrinsic and extrinsic apoptotic pathways in GC-1 spg cells.

### MP activated autophagy in GC-1 spg cells

**FIGURE 4:**
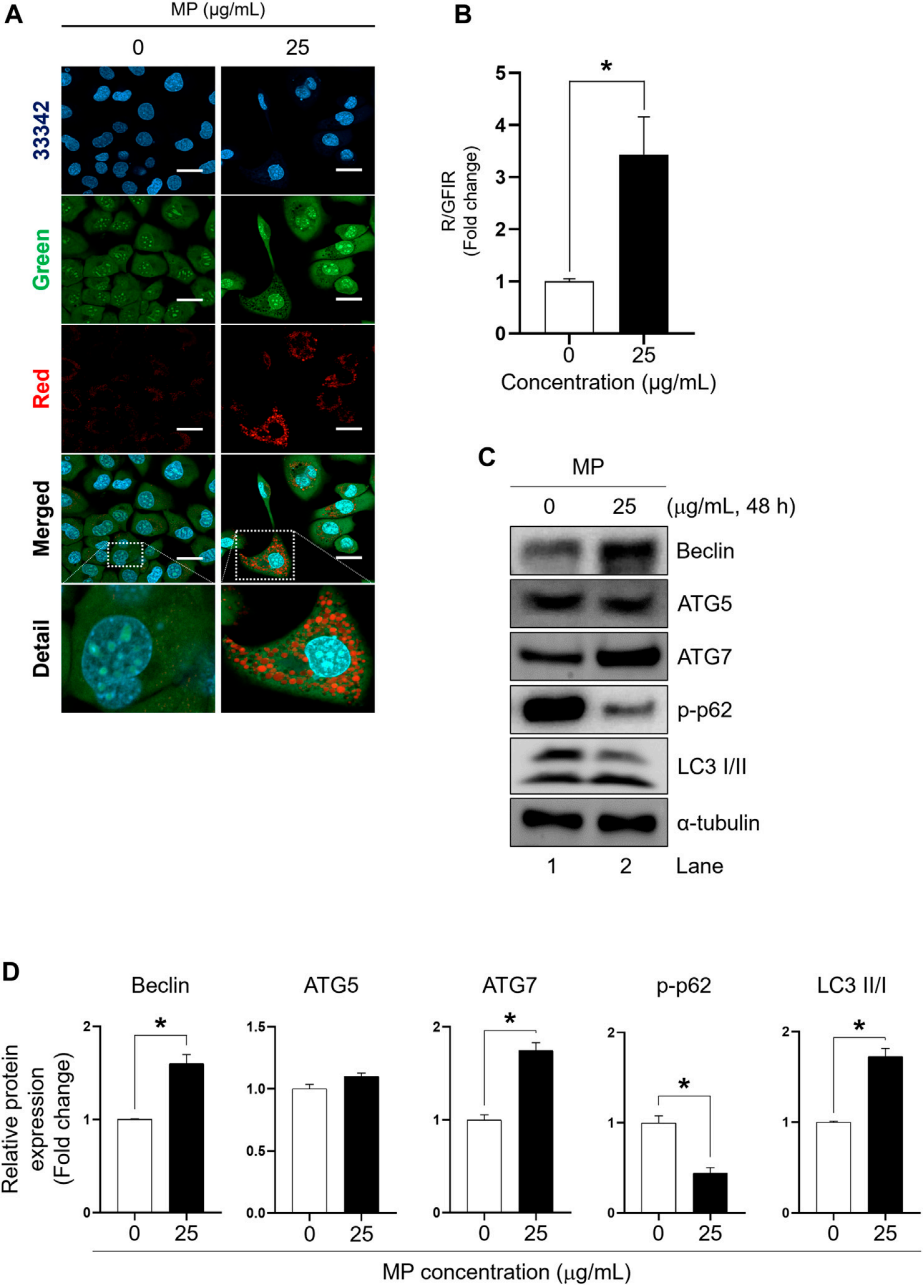
A mixture of PAEs (MP) stimulates autophagy in GC-1 spg cells. **(A)** Representative confocal microscopic images of acridine-orange (AO)-staining in GC-1 spg cells exposed to control (0 μg/mL) and MP treatments (25 μg/mL) (scale bar = 50 μm). **(B)** Red-to-green fluorescence intensity ratio (R/GFIR) shown in **(A)**. **(C)** Autophagy regulator protein: Beclin, an initiator of autophagy; ATG5 and ATG7, involved in autophagosome formation; and p62, which is transported into autophagosomes in GC-1 spg cells. **(D)** Quantified western blot data shown in **(C)**. Values are normalized to α -tubulin and presented as the mean ± SEM. The statistical analysis was performed using Student’s t-test, and a significant difference is indicated by an asterisk (*p < 0.05, *n* = 3). PAEs, phthalate esters; spg, spermatogonia; ATG5, autophagy-related 5; ATG7, autophagy-related 7; p-, phosphorylated; SEM, standard error of the mean.

The effect of MP on autophagy activation in GC-1 spg cells was assessed by examining acridine orange staining and the expression of autophagy regulator markers, including Beclin, autophagy-related 5 (ATG5), autophagy-related 7 (ATG7), p62, and LC3 proteins, which are specific to autophagy progression (Figure 4). The red-to-green fluorescence intensity ratio (R/GFIR) significantly increased in MP-exposed cells compared to the control (Figures 4A, B). While the expression of Beclin, ATG7, and LC3 II/I proteins notably increased in GC-1 spg cells exposed to 25 μg/mL MP, there was no change in the expression of ATG5 protein. Additionally, the phosphorylation of p62 protein, typically reduced during autophagy activation, significantly decreased (Figures 4C, D), suggesting that MP played a role in inducing autophagy in GC-1 spg cells.

### MP inhibited the phosphorylation of phosphoinositide 3-kinase (PI3K), protein kinase B, and mammalian target of rapamycin (mTOR) in GC-1 spg cells

**FIGURE 5:**
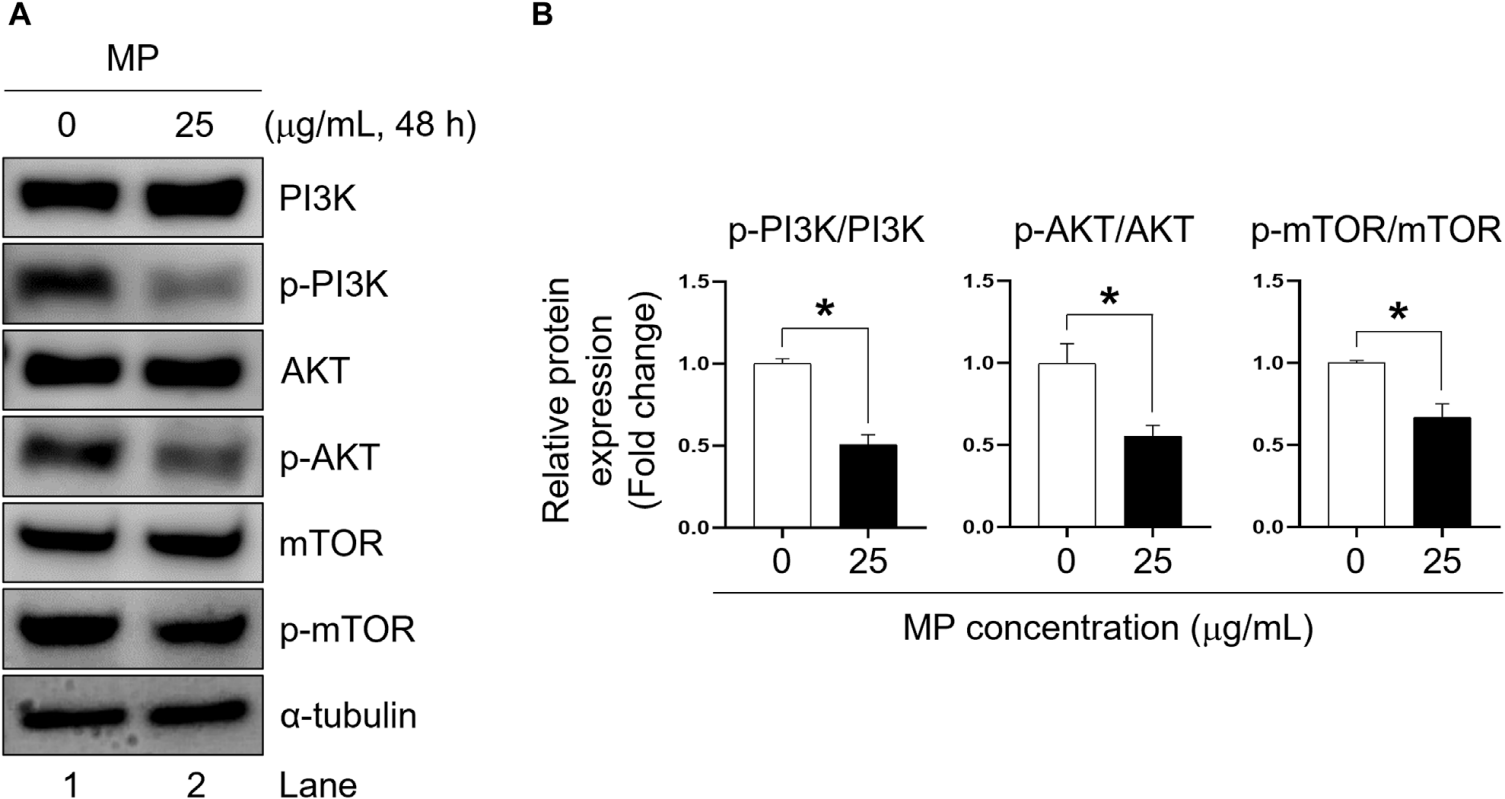
A mixture of PAEs (MP) blocks PI3K, AKT, and mTOR pathways in GC-1 spg cells. **(A)** Western blot images of PI3K-AKT-mTOR protein expression in GC-1 spg cells exposed to MP for 48 h. **(B)** Relative protein expression levels shown in **(A)**. The protein expressions are presented as mean ± SEM. The statistical significance was determined using Student’s t-test and is indicated by an asterisk (*p < 0.05, *n* = 3). PAEs, phthalate esters; spg, spermatogonia; PI3K, phosphoinositide 3-kinase; AKT, protein kinase B; mTOR, mammalian target of rapamycin; p-, phosphorylated; SEM, standard error of the mean.

To elucidate the impact of MP in GC-1 spg proliferation, we examined the phosphorylation levels of PI3K, AKT, and mTOR proteins. In GC-1 spg cells exposed to 25 μg/mL MP, phosphorylation of PI3K, AKT, and mTOR was significantly reduced compared to control cells (Figures 5A, B). These findings highlights the critical role of PI3K/AKT/mTOR signaling in regulating cell growth and survival, suggesting that MP-induced alteration in these pathways contribute to the disruption of GC-1 spg cell proliferation.

### Combination treatment alleviates MP-induced toxicity

**FIGURE 6:**
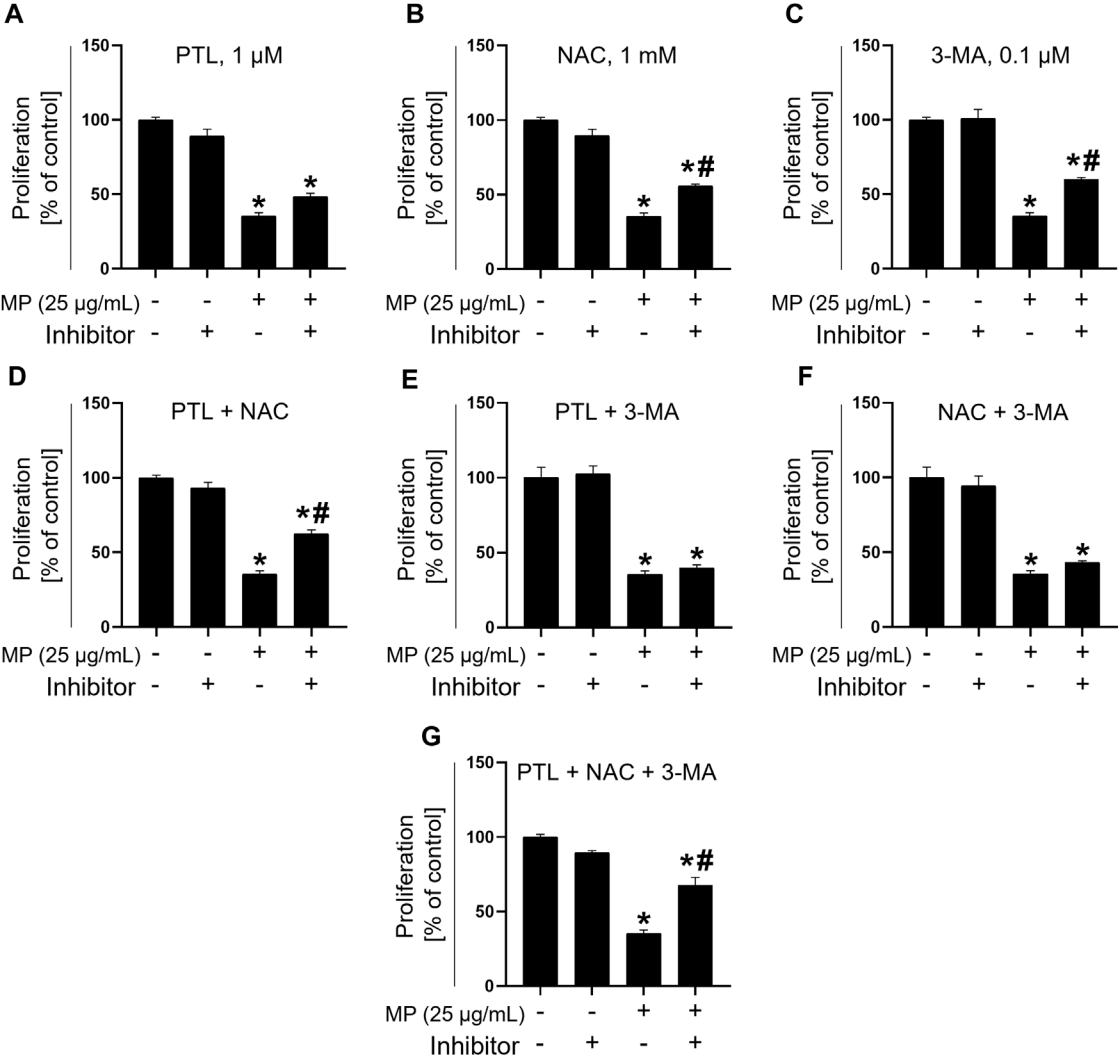
Combination treatment with PTL, NAC, and 3-MA mitigates toxicity induced by a mixture of PAEs (MP). **(A–C)** Proliferation rate addressing the mitigating effects of single inhibitors. **(D–F)** Alleviation using dual combination treatments. **(G)** Recovery using triple combination treatment. All data are presented as means ± SEM. Statistical significance was determined using one-way ANOVA with Tukey’s honestly significant difference; *p < 0.05, indicates a significant difference compared to the control and all experimental groups; #*p* < 0.05, indicates a significant difference between the 25 μg/mL MP co-treated with inhibitors and 25 μg/mL MP group (*n* = 3). PTL, parthenolide; NAC, N-acetylcysteine; 3-MA, 3-methyladenine; PAEs, phthalate esters; SEM, standard error of the mean; ANOVA, analysis of variance.

To assess the efficacy of inhibitors in alleviating MP-induced toxicity in GC-1 spg cells, a combination treatment involving three inhibitors − PTL, NAC, and 3-MA − was evaluated in single, dual, and triple combinations. Compared to the MP-exposed group (35.42%), cell proliferation increased non-significantly with PTL alone (48.44%), while both NAC (55.73%) and 3-MA (59.90%) substantially mitigated MP-induced mitigation of GC-1 spg cell proliferation (Figures 6A–C). In dual combinations, PTL + NAC showed marked proliferation recovery of proliferation (62.50%), while PTL + 3-MA (39.58%) and NAC + 3-MA (43.23%) exhibited similar effects (Figures 6D–F). The highest recovery was observed with the triple combination treatment (PTL + NAC + 3 MA, 67.71%), indicating synergistic mitigation of GC-1 spg cell proliferation (Figure 6G).

**FIGURE 7:**
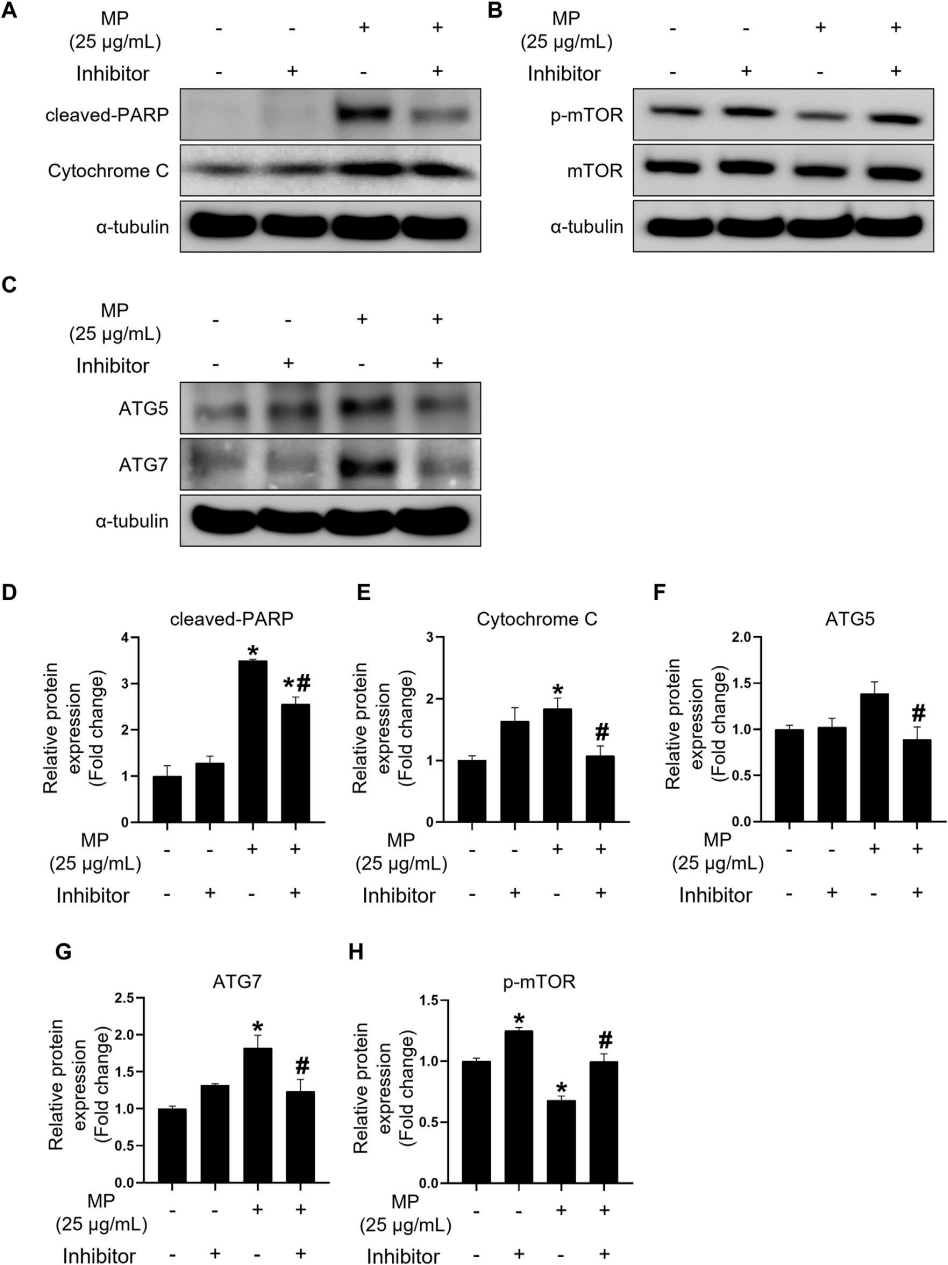
Combination treatment of PTL, NAC, and 3-MA alleviates apoptosis and autophagy induced by a mixture of PAEs (MP) and restores mTOR phosphorylation. **(A–C)** Representative western blot images of apoptosis (cleaved-PARP and cytochrome c), cell proliferation (p-mTOR), and autophagy (ATG5 and ATG7) protein expressions. **(D–H)** Normalized western blot images shown in **(A–C)**. Each protein expression is normalized to α-tubulin and presented as the mean ± SEM. The statistical analysis was performed using one-way ANOVA with Tukey’s honestly significant difference; *p < 0.05 indicates a significant difference between the control and all experimental groups; #*p* < 0.05 indicates a significant difference between the 25 μg/mL MP group with three inhibitors and the 25 μg/mL MP group (*n* = 3). PTL, parthenolide; NAC, N-acetylcysteine; 3-MA, 3-methyladenine; PAEs, phthalate esters; mTOR, mammalian target of rapamycin; PARP, poly (ADP-ribose) polymerase; p-mTOR, phosphorylated m-TOR; ATG5, autophagy-related 5; ATG7, autophagy-related 7; SEM, standard error of the mean; ANOVA, analysis of variance.

To investigate the effects of the triple inhibitors, we assessed the expression levels of proteins specific to apoptosis, autophagy, and proliferation regulation (Figures 7A–C). Treatment with the triple inhibitors led to a significant decrease occurred in the expression of intrinsic and execution apoptotic proteins, including cleaved-PARP and cytochrome c, respectively, compared to the MP control group (Figures 7D, E). Additionally, ATG5 and ATG7 protein levels were notably reduced under triple inhibitor treatment conditions (Figures 7F, G). Conversely, phosphor-mTOR levels significantly increased, reaching levels equivalent to those in the control (Figure 7H). These findings suggest that the triple combination treatment effectively restored cell proliferation by mitigating MP-induced apoptosis and autophagy.

## Discussion

In this study, we explored the toxicity of a mixed formulation of phthalate esters, comprising six phthalates with varying molecular weights: high- (DiNP), transitional- (DBP, BBP, and DEHP), and low- (DEP and DiBP) (Sedha et al., 2021). Various studies have detected several phthalates, including those examined in this study, in fingernails, foods, gloves, and house dust. A recent human biomonitoring study revealed that total phthalate concentrations ranged from 17.8 to 176 μg/g (median: 65.4 μg/g), with DEHP, DBP, and DiBP identified as the predominant compounds in fingernails (Li et al., 2022). Another study reported that dioctyl terephthalate, a replacement plasticizer of DEHP and DBP, was detected at the highest concentrations in foods (median = 2,510 μg/kg; max = 12,400 μg/kg) and gloves (28%–37% by weight) (Edwards et al., 2022). Additionally, research has found that BBP, DBP, DiNP, DEHP, and DiDP were present in house dust at concentrations ranging from 204 mg/kg to 3,360 mg/kg (Abb et al., 2009). Based on these studies, our aim was to mimic environmental exposure scenarios where individuals encounter multiple phthalates simultaneously. The MP proportion used in current study was selected to reflect environmentally relevant levels found in pregnant women’s urine (Zhou and Flaws, 2017; Amjad et al., 2021), and other *in vivo* experiments that have indicated adverse reproductive effects of the same MP in both males and females (Zhou et al., 2017a; Barakat et al., 2019). Various concentrations, both higher than the IC_50_ value (25 μg/mL) and those unaffected by MP (1.56 μg/mL), were assessed and revealed the synergistic inhibitory effect of phthalate esters as a mixture (Supplementary Figure S2).

Single PAE-induced oxidative stress is known to induce apoptosis and autophagy (Scherz-Shouval and Elazar, 2011; Wu and Bratton, 2013; Feng et al., 2017). The MP-induced ROS generation was markedly revealed using DCFDA staining, with the DCF fluorescence signal mainly localized in the cytoplasm, as previously reported (Afri et al., 2004; Shen et al., 2013). Annexin V staining using flow cytometry further indicated MP-induced apoptosis of GC-1 spg cells, suggesting that concurrent excessive cellular ROS production and apoptosis are crucial factors in MP-induced male germ cell toxicity.

To understand MP-induced apoptosis modulation in GC-1 spg cells, we evaluated apoptosis pathway regulator proteins. First, intrinsic apoptotic regulator proteins, which are regulated by intracellular cell death signals in a mitochondria-dependent manner (Pfeffer and Singh, 2018), showed significant changes. MP substantially increased the expression levels of BAX, cytochrome c, and caspase-9, while BCL2 expression remained unchanged. The elevated BAX/BCL2 ratio indicates a pro-apoptotic environment induced by MP, leading to cytochrome c release and caspase-9 cleavage. Second, extrinsic apoptosis, activated when external ligand binding to death receptors (Jan and Chaudhry 2019), showed marked increases in FAS and cleaved caspase-8 levels in MP-exposed GC-1 spg cells. Both apoptotic pathways ultimately activated executioner proteins, namely caspase-3, -7, and cleaved-PARP, suggesting that MP suppressed the proliferation of GC-1 spg cells by simultaneously engaging intrinsic and extrinsic apoptotic pathways.

Although autophagy primarily helps maintain cellular homeostasis mainly by degrading and recycling damaged organelles, misfolded proteins, and other malfunctioning cellular components (Glick et al., 2010), it is closely related to apoptosis and can induce cell death. Proteins involved in autophagy, such as Beclin-1, can participate in processes occurring during apoptosis (Rikiishi, 2012). PAEs are reported to induce autophagy in male germ cells (Zhang et al., 2016; Valenzuela-Leon and Dobrinski, 2017; Sun et al., 2018), and our data indicated that MP induced autophagy. This was evidenced by the upregulation of Beclin, ATG7, LC3 II/I, and the downregulation of p62, all crucial in autophagy progression (Tanida, 2011; Meyer et al., 2013; Xiong, 2015; Changotra et al., 2022). Although ATG5, a pivotal player in both apoptosis and autophagy, showed slightly increased expression levels, the difference was not statistically significant, suggesting that MP-induced autophagy may be dependent on ATG7, as observed in previous research (Walls et al., 2010; Rikiishi, 2012; Zhang et al., 2015). Acridine orange staining, useful for detecting autophagy, showed substantial red fluorescence in MP-exposed cells, indicating increased autophagic activity (Thomé et al., 2016). These results collectively demonstrate that MP induces autophagy and hinders the proliferative activity of male germ cells.

The PI3K-AKT pathway influences mTOR activity (Kim and Guan, 2019), playing pivotal role in regulating essential cellular processes such as cell growth, metabolism, survival, proliferation, and angiogenesis in male reproductive function (Cho, 2014; Deng et al., 2021). Moreover, this signaling pathway is known to inhibit apoptosis (Yao et al., 2014), with its inhibition potentially promoting the mitochondrial apoptosis pathway (Fulda, 2014; Yao et al., 2020). In addition, the PI3K/AKT/mTOR pathway serves as a major regulator of autophagy, primarily through the inhibition of the autophagic process, with mTOR acting as a central inhibitor of autophagy (Heras-Sandoval et al., 2014). Consequently, exposure to MP substantially suppressed the phosphorylation of PI3K, AKT, and mTOR, suggesting that MP inhibits this signaling pathway by directly impeding key kinase activity, thereby hindering the proliferation of GC-1 spg cells.

While MP adversely affected cellular activities such as ROS generation, apoptosis, autophagy, and proliferation, NAC, PTL, and 3-MA demonstrated remarkable efficacy in restoring GC-1 spg cell proliferation from MP-induced damage. These inhibitors are known to counteract oxidative stress, apoptosis, and autophagy-induced toxicants (Mao and Zhu, 2018; Gan et al., 2020; Liu et al., 2020). We evaluated NAC, PTL, and 3-MA individually, in dual combinations, and in a triple combination to mitigate MP-induced damage in GC-1 spg cells and facilitate recovery. In single inhibitor treatments, PTL did not show a recovery effect, whereas NAC and 3-MA treatments exhibited recovery effects. Dual combination treatments with PTL + NAC significantly alleviated proliferation damage due to significant inhibition of cleaved-PARP (Supplementary Figure S3). Although the NAC + 3-MA treatment group displayed a significant decrease in cleaved-PARP protein levels, unlike the PTL + 3-MA group, both treatment combinations did not significantly alleviate proliferation damage in MP-exposed GC-1 spg cells. This indicates that reducing oxidative stress and inhibiting NF-κB can protect GC-1 spg cells. However, inhibiting autophagy with 3-MA counteracts the protective effects of NAC, resulting in continued cell death despite the inhibition of apoptosis. The triple combination treatment of inhibitors demonstrated the most substantial recovery effect. Given the composition of six PAEs in MP, these findings suggest that each inhibitor acted complementarily on MP-induced cell damage, leading to a synergistic recovery effect. Furthermore, the triple combination treatment significantly reduced the expression levels of cleaved-PARP, cytochrome c, ATG5, and ATG7, while restoring mTOR expression to a level similar to that of the control, suggesting that MP-induced damage can be effectively mitigated using the triple inhibitor combination treatment.

Current study demonstrated the dose-response impact of MP-induced male germ cell toxicity, involving ROS generation, apoptosis, autophagy, and suppression of proliferation regulator protein expression, highlighting the utility of GC-1 spg cells in evaluating environmental pollutants’ effect on male germ cell toxicity. In addition, single PAEs like DEP, DEHP, and DBP have been shown to damage the blood–testis barrier (Kumar et al., 2015; Yang et al., 2022; Ma et al., 2023), indicating their potential adverse impact on Sertoli cells. As a mixture containing these PAEs, MP induced marked reduction of zonula occludens-1 protein expression in TM4 (Sertoli cells) in this study (Supplementary Figure S4), suggesting its systemic testicular toxicity, in addition to the effects of MP on type B spermatogonia. Therefore, further *in vivo* study is warranted to elucidate the comprehensive adverse impacts on other testicular cells, such as Sertoli cells, Leydig cells, endothelial cells, and spermatogonial stem cells. Importantly, however, our study provided the experimental evidence of the effectiveness of chemical inhibitors as single or combined formulas in protecting male germ cells from MP-induced toxicity, contributing to the protection against environmental toxins like MP exposure.

## Conclusion

This study demonstrated that MP impeded the growth of GC-1 spg cells by inducing ROS generation, apoptosis, autophagy, and suppressing key kinases, PI3K, AKT, and mTOR. Remarkably, a triple inhibitor combination treatment comprising NAC, PTL, and 3-MA significantly alleviated MP-induced germ cell toxicity, reinstating proliferation, diminishing autophagy and apoptosis, and restoring mTOR phosphorylation. These findings offer valuable insights into the cellular mechanisms underlying MP-induced male germ cell toxicity and provide crucial data for potential intervention measures aimed at mitigating toxicity induced by multiple PAEs.

## Figures and Tables

**Table:** 

**AKT**	protein kinase B
**ANOVA**	analysis of variance
**AO**	acridine orange
**APC**	allophycocyanin
**ATG5**	autophagy-related 5
**ATG7**	autophagy-related 7
**BAX**	BCL2-associated X protein
**BBP**	benzyl butyl phthalate
**BCL2**	B-cell lymphoma 2
**DBP**	dibutyl phthalate
**DCF**	dichlorodihydrofluorescein
**DCFDA**	dichlorodihydrofluorescein diacetate
**DEHP**	bis (2-ethylhexyl) phthalate
**DEP**	diethyl phthalate
**DiBP**	diisobutyl phthalate
**DiNP**	diisononyl phthalate
**DMEM**	Dulbecco’s modified Eagle’s medium
**DMSO**	Dimethyl sulfoxide
**DPBS**	Dulbecco’s phosphate buffered saline
**EDCs**	endocrine-disrupting chemical
**EDTA**	ethylenediaminetetraacetic acid
**FACS**	fluorescence-activated cell sorting
**FBS**	fetal bovine serum
**IC50**	half maximal inhibitory concentration
**3-MA**	3-methyladenine
**MP**	mixture of PAEs
**mTOR**	mammalian target of rapamycin
**NAC**	N-acetylcysteine
**PAEs**	phthalate esters
**PARP**	poly (ADP-ribose) polymerase
**PBS/FBS**	DPBS + 1% FBS
**PBS-T**	DBPS + 0.1% Tween 20
**PI**	propidium iodide
**PI3K**	phosphoinositide 3-kinase
**PTL**	parthenolide
**R/GFIR**	red-to-green fluorescence intensity ratio
**ROS**	reactive oxygen species
**RT**	room temperature
**spg**	spermatogonia
**TORC1**	mTOR complex 1

## Data Availability

The original contributions presented in the study are included in the article/Supplementary Material, further inquiries can be directed to the corresponding author.
