# Supplementary material for: Elucidating the mechanisms and mitigation strategies for six-phthalate-induced toxicity in male germ cells
Source: Front Cell Dev Biol. 2024 Jul 10;12:1398176. doi: 10.3389/fcell.2024.1398176 (PMC11266291; doi:10.3389/fcell.2024.1398176)
Supplement: Supplementary file 1 [file DataSheet1.docx]

**Supplementary Materials**

**Elucidating the Mechanisms and Mitigation Strategies for Six-Phthalate-Induced Toxicity in Male Germ Cells**

Seok-Man Kim^1^, Yong-Hee Kim^2^, Gil Un Han^1^, Seul Gi Kim^1^, Bang-Jin Kim^3^, Sung-Hwan Moon^1^, Seung Hee Shin^1^, Buom-Yong Ryu^1*^

^1^Department of Animal Science and Technology, Chung-Ang University, Anseong-Si, Gyeonggi-Do, 17546, Republic of Korea

^2^AttisLab Inc., Anyang-Si, Gyeonggi-Do, 14059, Republic of Korea

^3^Department of Surgery, Division of Surgical Sciences, Columbia University Irving Medical Center, New York, NY 10032

*Corresponding author: Buom-Yong Ryu, Ph.D., Department of Animal Science and Technology, Chung-Ang University, Anseong-Si, Gyeonggi-Do 17546, Republic of Korea; E-mail, byryu@cau.ac.kr; Tel, 82-31-670-4687; Fax, 82-31-676-0062.

**Supplementary methods**

**Sulforhodamine B assay**

The GC-1 spg cells were seeded at a density of 2 × 10^3^ cells per well in a 96-well plate and incubated for 24 h. Subsequently, the cells were treated with MP and incubated for an additional 48 h. After the desired treatment period, the culture medium was carefully removed. The cells were fixed by adding 10% ice-cold trichloroacetic acid (T6399, Sigma-Aldrich, St. Louis, MO, USA) directly to the culture plates and the plates were incubated at 4°C for 4 h. The fixative solution was discarded and the fixed cells were washed with distilled water to remove any excess fixative. After the plates were sufficiently air-dried, sulforhodamine B solution [230162, Sigma-Aldrich, a 0.4% (w/v) sulforhodamine B dye solution in 1% acetic acid] was added to cover the cells in each well of the culture plates. The plates were then incubated for 30 min at room temperature (RT; 20–25°C) and protected from light. Following staining, the excess dye solution was carefully removed by washing the plates with 1% acetic acid solution, and the plates were allowed to air dry completely. Once dry, 10 mM Tris buffer (pH 10.5) was added to each well to solubilize the bound dye, ensuring that the volume was sufficient to cover the stained cells. The plates were gently rocked for 15 min to ensure complete solubilization of the dye. Finally, the solubilized dye solution was transferred to a clear microplate, and the absorbance of each well was measured at 570 nm using a microplate reader (Spectramax190, Molecular Device, USA).

**Supplementary Figure Legends**

**Supplementary Figure S1. Cytotoxicity assessment of GC-1 spg cells exposed to MP using a sulforhodamine B assay.** (**A**) Graphical representation of proliferation in GC-1 spg cells exposed to MP for 48 h. All values are represented as mean ± SEM. Statistical significance was evaluated using Dunnett’s test and is denoted by an asterisk (**p* < 0.05, n = 3). NS, non-significant; MP, mixture of phthalate esters; spg, spermatogonia, SEM, standard error of mean

**Supplementary Figure S2. Cytotoxicity evaluation of GC-1 spg cells exposed to single PAEs using a sulforhodamine B assay.** (**A–F**) Graphical representation of proliferation in GC-1 spg cells exposed to DEP, DEHP, DBP, DiNP, DiBP, and BBP for 48 h. Considering the ratio of each phthalate to MP (25 µg/ml), the calculated concentrations were: DEP (35%, 8.75 µg/ml), DEHP (21%, 5.25 µg/ml), DBP (15%, 3.75 µg/ml), DiNP (15%, 3.75 µg/ml), DiBP (8%, 2.00 µg/ml), and BBP (5%, 1.25 µg/ml). All values are expressed as mean ± SEM. Statistical significance was assessed using Dunnett’s test and is indicated by an asterisk (**p* < 0.05, n = 3). NS, non-significant; MP, mixture of phthalate esters; spg, spermatogonia, SEM, standard error of mean; DEP, diethyl phthalate; DEHP, di(2-ethylhexyl) phthalate; DBP, di-n-butyl phthalate; DiNP, diisononyl phthalate; DiBP, diisobutyl phthalate; BBzP, butyl benzyl phthalate

**Supplementary Figure S3. Cleaved-PARP protein expression of dual inhibitor combination treatment in MP-exposed GC-1 spg cells.** (**A**) Representative western blot images of each dual inhibitor combination treatment (PTL + NAC, 3-MA + PTL, and 3-MA + NAC) in GC-1 spg cells exposed to control (0 μg/mL) and MP treatments (25 μg/mL) are shown. (**B**) Graphical representation of quantified western blot data shown in (**A**). All values are expressed mean ± SEM. Statistical significance was performed using Dunnett’s test and (control and control + inhibitors) and (MP and MP + inhibitors) were compared; **p* < 0.05, n = 3. MP, mixture of phthalate esters; spg, spermatogonia; SEM, standard error of mean; PTL, parthenolide; 3-MA, 3-methyladenine; NAC, N-acetylcysteine

**Supplementary Figure S4. ZO-1 protein expression level in Sertoli cells (TM4) after MP exposure.** (**A**) Representative western blot band images in Sertoli cells (TM4) exposed to MP and the control for 48 h. (**B**) Graphical representation of quantified ZO-1 protein shown in (**A**). All values are expressed as mean ± SEM. Statistical significance was performed using Student’s *t*-test and is denoted by an asterisk (**p* < 0.05, n = 3). MP, mixture of phthalate esters; spg, spermatogonia; SEM, standard error of mean

**Figure S1**

**
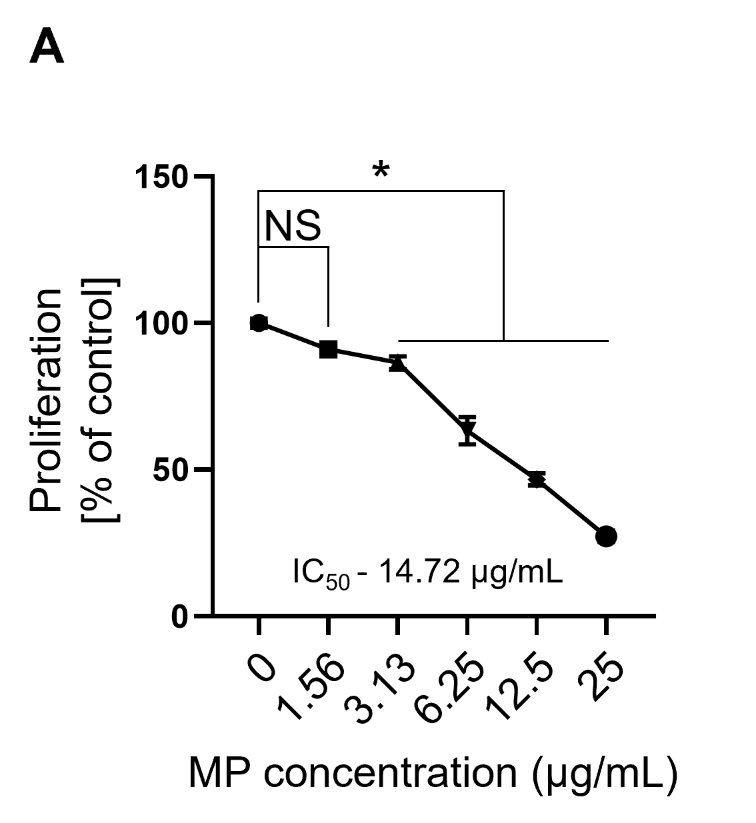
**

**Figure S2**

**
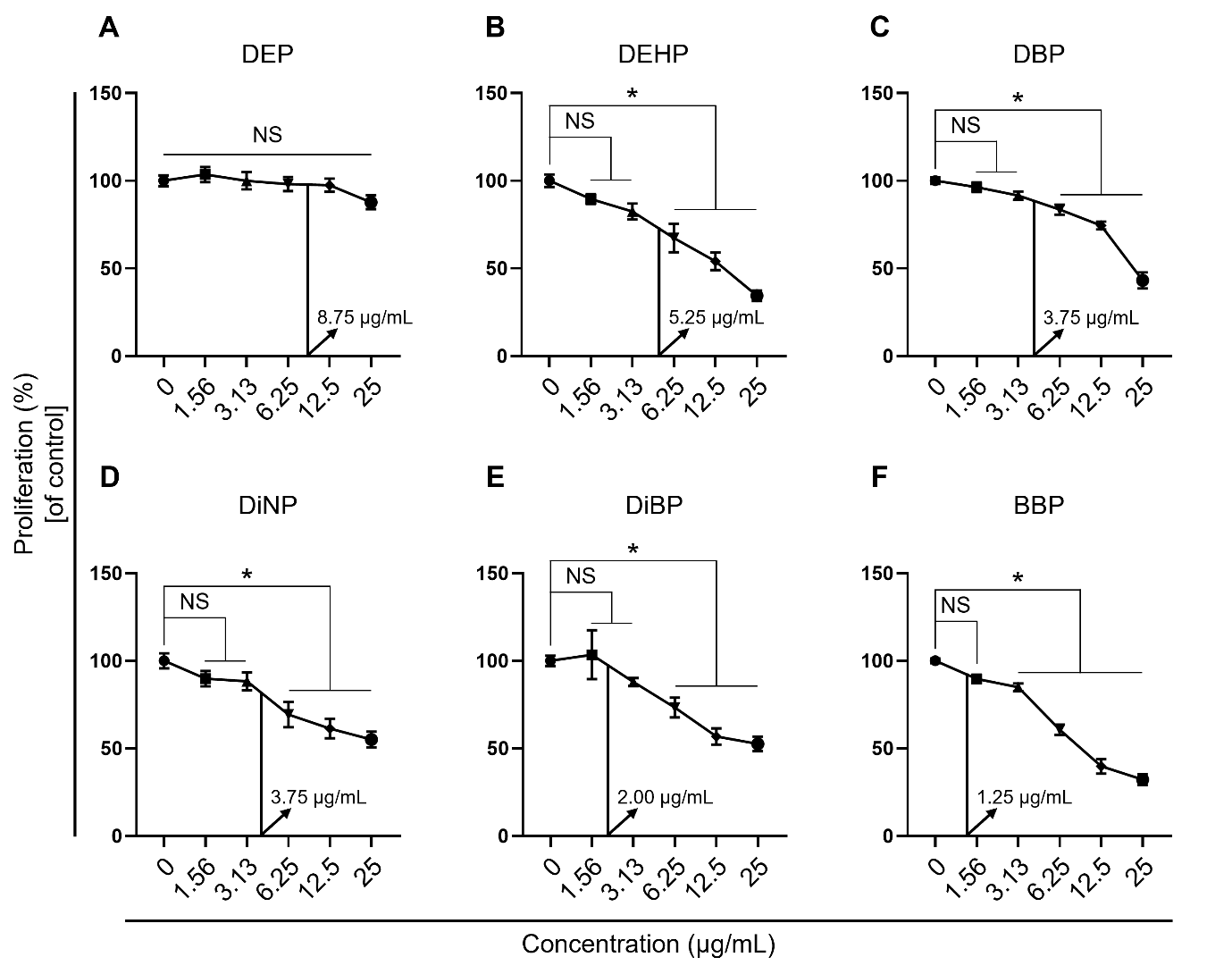
**

**Figure S3**

**
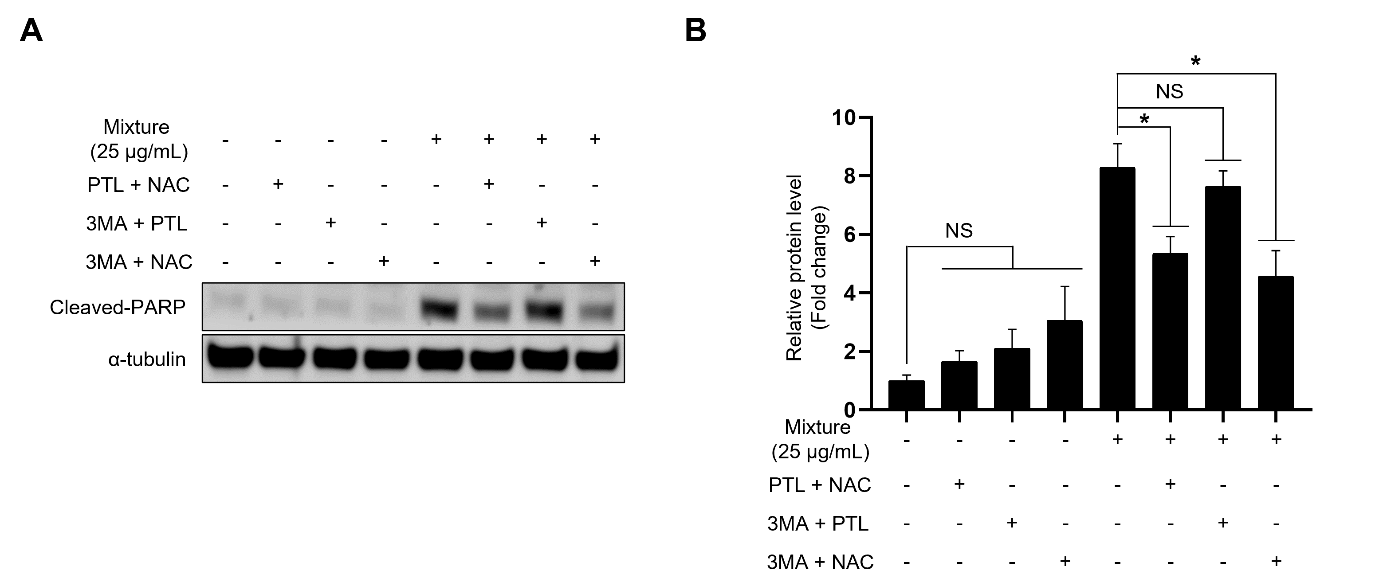
**

**Figure S4**

**
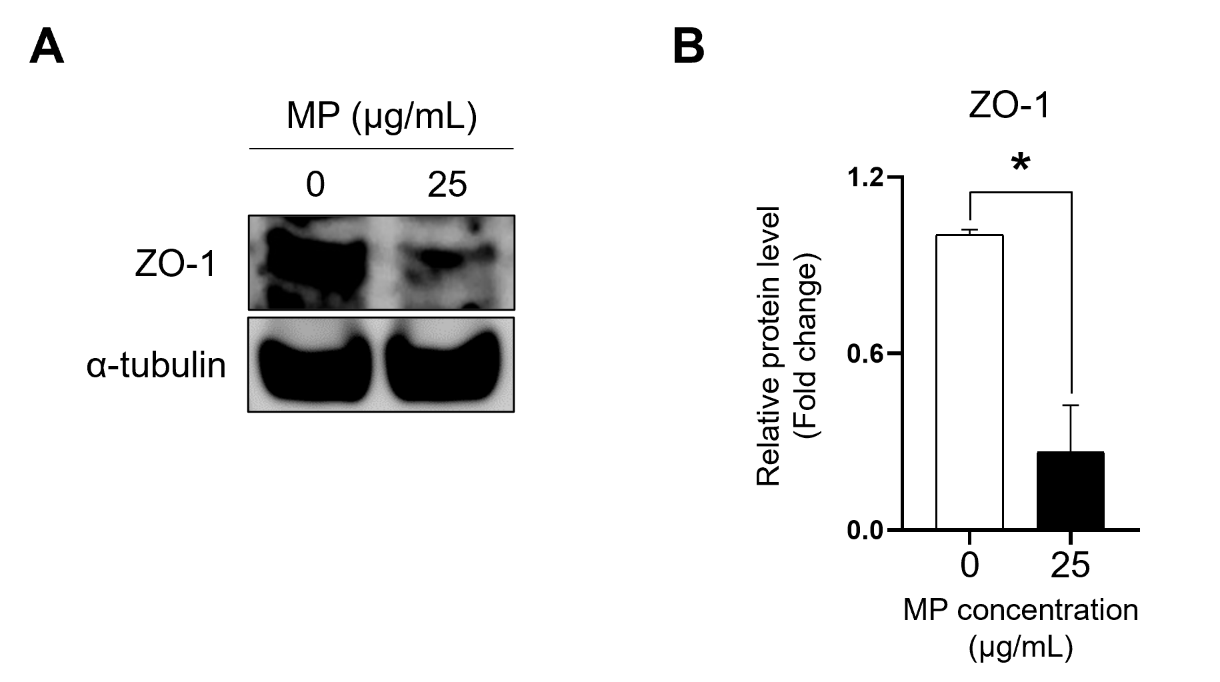
**
